# Autophagic Clearance of Lipid Droplets Alters Metabolic Phenotypes in a Genetic Obesity–Diabetes Mouse Model

**DOI:** 10.1007/s43657-022-00080-z

**Published:** 2022-11-19

**Authors:** Ningxie Chen, Boxun Lu, Yuhua Fu

**Affiliations:** State Key Laboratory of Medical Neurobiology and MOE Frontiers Center for Brain Science, School of Life Sciences, Fudan University, Shanghai, 200438 China

**Keywords:** Lipid droplets, Autophagy, Obesity–diabetes, LD·ATTECs, Targeted degradation, Metabolic cages

## Abstract

Lipid droplets (LDs) are intracellular organelles that store neutral lipids, and their aberrant accumulation is associated with many diseases including metabolic disorders such as obesity and diabetes. Meanwhile, the potential pathological contributions of LDs in these diseases are unclear, likely due to a lack of chemical biology tools to clear LDs. We recently developed LD-clearance small molecule compounds, Lipid Droplets·AuTophagy TEthering Compounds (LD·ATTECs), that are able to induce autophagic clearance of LDs in cells and in the liver of *db/db* (C57BL/6J Lepr^db^/Lepr^db^) mouse model, which is a widely used genetic model for obesity–diabetes. Meanwhile, the potential effects on the metabolic phenotype remain to be elucidated. Here, using the metabolic cage assay and the blood glucose assay, we performed phenotypic characterization of the effects of the autophagic degradation of LDs by LD·ATTECs in the *db/db* mouse model. The study reveals that LD·ATTECs increased the oxygen uptake of mice and the release of carbon dioxide, enhanced the heat production of animals, partially enhanced the exercise during the dark phase, decreased the blood glucose level and improved insulin sensitivity. Collectively, the study characterized the metabolic phenotypes induced by LD·ATTECs in an obesity–diabetes mouse model, revealing novel functional impacts of autophagic clearance of LDs and providing insights into LD biology and obesity–diabetes pathogenesis from the phenotypic perspective.

## Introduction

Obesity is the most frequently outbroke metabolic disease in the world, with a rapid increasing incidence and prevalence. Obesity is also positively associated with comorbidities, including type 2 diabetes mellitus (T2DM) and hypertension (Gross et al. 2017). Studies previously showed that 60–90% of all patients with T2DM are or have been obese (after called obesity–diabetes) (Stumvoll et al. 2005). Obesity–diabetic patients suffer from both obesity and insulin resistance (Guilherme et al. 2008).

Obesity–diabetes is associated with excess lipids, accompanied by excessive lipid droplets (LDs) in adipose and non-adipose tissues (Gluchowski et al. 2017; Nakamura and Sadoshima 2020), and adipose tissue is also a vital endocrine organ that modulates energy homeostasis (Zhang et al. 1994). LDs are intracellular organelles that store neutral lipids in various cell types, especially adipocytes and hepatocytes (Olzmann and Carvalho 2019). LDs regulate lipid storage and metabolism, and they are involved in various diseases including cancer (Hoy et al. 2021), obesity (Engin 2017), and diabetes (Bosch et al. 2020), which are characterized by the excessive accumulation of LDs in different cell types. LDs are traditionally considered as neutral lipid reservoirs, which protect cells from lipid toxicity caused by excess free fatty acids (FFAs) (Auclair et al. 2018; Laurens et al. 2016; Schley et al. 2020). Meanwhile, degradation of LDs with a concomitant reduction of FFAs ameliorates disease phenotype (Abu-Gazala et al. 2018; Fu et al. 2021; Wang et al. 2014; Xu et al. 2014). For example, the removal of cellular LD accumulation levels is associated with improvement of blood glucose in *db/db* mouse model (Abu-Gazala et al. 2018; Jo et al. 2021; Mizusaki et al. 2019). Thus, the potential causal relationship between excessive LDs accumulation and pathogenic phenotype has not been established and remains controversial, partially due to a lack of chemical tools to clear LDs. From a broader perspective, the phenotypic changes induced by LDs lowering have not been characterized, especially in the obesity–diabetes context. Characterization of such phenotypes may provide valuable information for the phenome of LDs and novel insights into the lipid biology.

Such characterization becomes feasible after our recent invention of small molecule compounds that specifically clear LDs (Fu et al. 2021). We designed and synthesized Lipid Droplets·AuTophagy TEthering Compounds (LD·ATTECs), which are compounds that tether LDs to the autophagosomes for autophagic degradation, a well-established mechanism of LD clearance (Singh et al. 2009). In this study, we characterized the phenotypic effects of LD·ATTECs in the *db/db* mouse model by metabolic cage experiments and blood glucose measurement to obtain a comprehensive understanding of the LDs’ contribution to the metabolic phenotypes.

## Materials and Methods

### Compounds and Metabolic Cage Experiments

Compounds C3 and C4 were designed and synthesized as previously published (Fu et al. 2021). Mice were maintained at the Shanghai Model Organisms facility. Mice were group-housed (three to five adult mice per cage) in individually vented cages with a 12 h light/dark cycle. The mouse experiments were performed following the Animal Research: Reporting of In Vivo Experiments (ARRIVE) guidelines and all relevant ethical regulations. The protocol used in animal experiments was approved by The Animal Care and Use Committee of the Shanghai Model Organisms facility. For *db/db* mouse experiments, 19-week-old *db/db* male mice (Hummel et al. 1966) were obtained at Shanghai Model Organisms. The mice were on ad libitum access to standard chow and water. The mice were randomly divided into three groups with seven mice in each group for i.p. injections: (1) DMSO group: 1% DMSO + 39% PEG300 + 5% Tween-80 + 55% H_2_O (DMSO vehicle), (2) C3 group: LD·ATTEC3 in DMSO vehicle, (3) C4 group: LD·ATTEC4 in DMSO vehicle. For groups one to three, compounds (150 μL) were injected to reach 30 mg/kg for each mouse. Compounds were administered once a day by i.p. injections for 2 weeks, and metabolic cage experiments were done during the last 3 days. Whole-body oxygen consumption (VO_2_) and carbon dioxide production (VCO_2_) were measured using CLAMS (CLAMS, Columbus Instruments).

The metabolic cage instrument is powered on and warmed up for at least 30 min. Put the cage with bedding on the metabolic cage frame, after weighting the water, put the food trough and drinking equipment on the metabolic cage cover. Start the computer and open the Oxymax software, the software automatically scans the link status of the hardware, the hardware is running well, and the hardware detection window is automatically minimized; open the ternary gas cylinder, perform gas calibration, close the gas bottle and weigh the mice, and put it into the corresponding metabolic cage. Enter the mouse information of the corresponding cage position and the experimental data storage path in the experimental settings, and then start the detection. The detection time is 72 h. During the experiment of the metabolic cage, make sure that the changes of light and ambient temperature is normal, and ensure that the room of the metabolic cage is well ventilated, and minimize human interference and the interference of the surrounding environment during the day. After the metabolic cage experiment, the mice were weighed again, and the remaining amount of drinking water was measured. VO_2_ and VCO_2_ were measured at 13 min intervals during the dark phase for 12 h. Respiratory exchange ratio (RER) was calculated from the VO_2_ and VCO_2_ values, and energy expenditure (heat formation[(3.815 + 1.232 × RER) × VO_2_ (in liters)] was calculated as previously described (Choi et al. 2015).

For body weight assay, wild-type mice (19-week-old) were injected with LD·ATTECs and weighted body weight each day during two weeks, the method and concentration are the same as the metabolic cage. The protocol used in animal experiments was approved by The Animal Care and Use Committee of Shanghai Medical College of Fudan University (Approval #202004001S).

### Glucose Tolerance Test

Mice (mice for metabolic cage assay) were fasted for overnight and injected intraperitoneally (i.p) with glucose (Cat. No. 63005518, Sinopharm Group) (2 g/kg body weight) in sterile water. Blood samples were taken before the glucose administration and then at 15, 30, 60 and 120 min after injection. Blood glucose levels were measured by a glucometer (Performa Excellent, Roche Company).

### Insulin Tolerance Test

BKS-db mouse (BKS-Leprem2Cd479/Gpt) was ordered from Gempharmatech Co., Ltd (Cat. No. T002407). Mice were fasted for overnight and injected intraperitoneally (i.p) with insulin 2 U kg^−1^ body weight (Cat. No. 40112ES25, Yeasen Biotechnology (Shanghai) Co., Ltd.). Blood samples were taken before the glucose administration and then at 30, 60, 90 and 120 min after injection. Blood glucose levels were measured by a glucometer (Bayer HealthCare LLC).

### Analysis and Statistics

All statistical analyses were performed using GraphPad Prism 8 (GraphPad Software). Results are presented as the mean ± SEM of all data points. All data were tested using one-way ANOVA or two-way ANOVA as described in the figure legends. Statistical significance was considered at *p* value < 0.05.

## Results

### LD·ATTECs Ameliorated the Respiratory Phenotype in db/db Mice

**Fig. 1:**
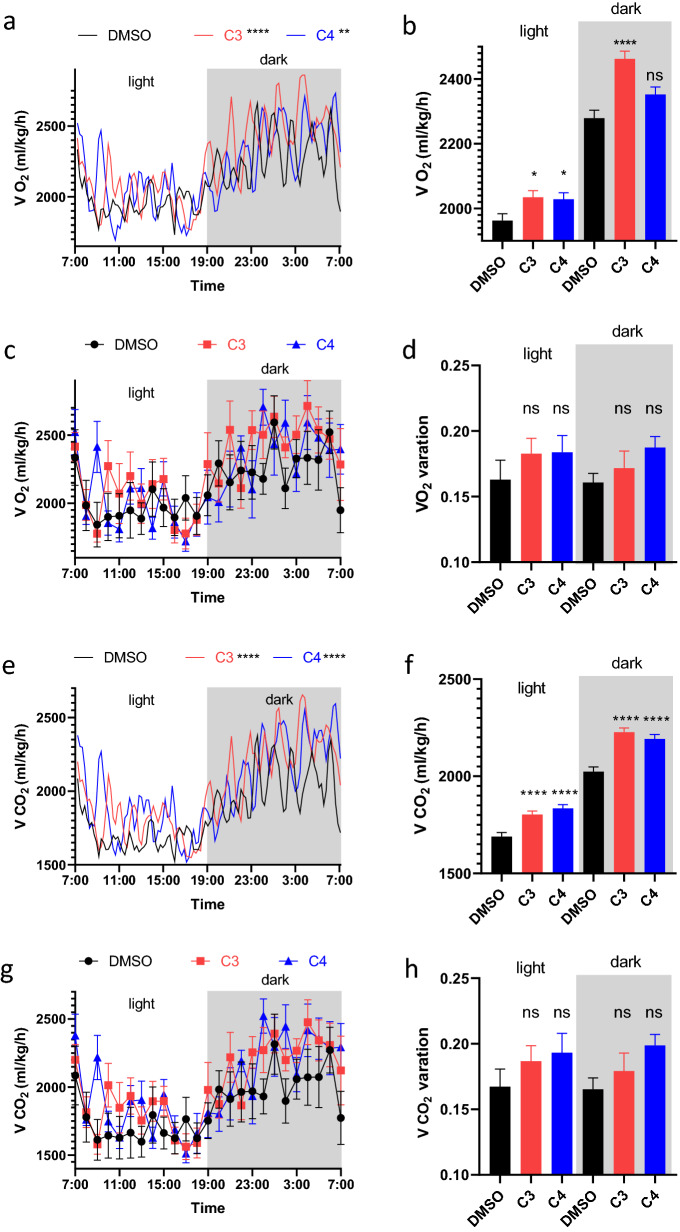
LD·ATTECs affect respiratory phenotypes in *db/db* mice. **a**,** b** Whole-body oxygen consumption of mice during the day phase (12 h) and dark phase (12 h) (all data points). **c** Whole-body oxygen consumption of mice during the day phase (12 h) and dark phase (12 h) (hourly data points). **d** Coefficient of variation analysis of the individual total oxygen consumption. **e**, **f** Whole-body carbon dioxide production of mice during the day phase (12 h) and dark phase (12 h) (all data points). **g** Whole-body carbon dioxide production of mice during the day phase (12 h) and dark phase (12 h) (hourly data points). **h** Coefficient of variation analysis of the individual carbon dioxide production. *N* = 7 per group, data are mean ± SEM. *p value < 0.05; **p value < 0.01; ****p value < 0.0001; ns (non-significant), *p* value > 0.05. One-way ANOVA (**b**, **d**, **f**, **h**), two-way ANOVA (**a**, **e**)

**Fig. 2:**
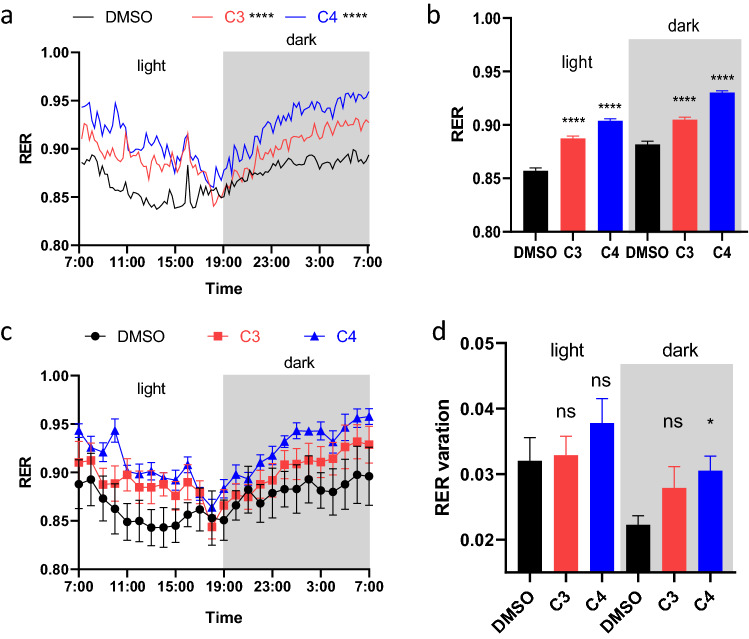
LD·ATTECs affect the respiratory exchange ratio in *db/db* mice. **a**, **b** RER of mice during the day phase (12 h) and dark phase (12 h) (all data points). **c** RER of mice during the day phase (12 h) and dark phase (12 h) (hourly data points). **d** Coefficient of variation analysis individual RER. *N* = 7 per group, data are mean ± SEM. *p value < 0.05; ****p value < 0.0001; ns, *p* value > 0.05. One-way ANOVA (**b**, **d**), two-way ANOVA (**a**)

To explore the effect of LD·ATTECs (LD·ATTEC3, C3; LD·ATTEC 4, C4) on respiratory phenotype in *db/db* mice (Ni et al. 2022; Trayhurn 2017), we intraperitoneally injected the *db/db* mice with LD·ATTECs at 30 mg/kg per day versus the DMSO control for 14 days. The mice were then tested by the metabolic cage experiments started on day ten. Both C3 and C4 significantly increased oxygen consumption than the DMSO control group (Fig. 1a–c), indicating that mice needed more aerobic for respiratory. The coefficient of the oxygen consumption variation of each individual mouse during light or dark phase was slightly increased in the C3 or C4-injected groups (Fig. 1d), indicating the C3 or C4-injection led to non-significant individual fluctuation increase during test period, possibly due to different adaptations to the compound injections. We also analyzed the carbon dioxide production based on metabolic cage data, and we observed significantly increased carbon dioxide production in mice injected with compound C3 or C4 (Fig. 1e–g), which is consistent with the higher respiratory suggested by the elevated oxygen consumption. Besides, the coefficient of carbon dioxide production variation of each individual mouse was also marginally higher in the C3 or C4-injected groups compared with the DMSO-injected control group (Fig. 1h). The ratio of produced CO_2_ to consumed O_2_, i.e., the RER is an indicator of the type of fuel (lipids vs. glucose) that is being metabolized (Gupta et al. 2017; Haugen et al. 2007; Ni et al. 2022). The C3 or C4-injected mice displayed higher RER compared to the DMSO-injected control mice (Fig. 2a–c). The increased RER suggests increased ratio of utilizing glucose as the fuel. This is intriguing because the degradation of LDs likely trigger utilizing more lipids as the fuel. The C3 or C4-injection changed the RER in *db/db* mice towards the wild-type direction, suggesting a rescue effect correcting the aberrant RER (Choi et al. 2015; Osborn et al. 2010). The C3 or C4-injection also increased the coefficient of individual RER variation (Fig. 2d), indicating that LD·ATTECs possibly shifted the body cells to normal metabolism. Collectively, LD·ATTECs enhanced oxygen consumption and carbon dioxide production with a concomitant higher RER. These results demonstrate that LD·ATTECs improved the respiratory phenotype in the *db/db* mice.

### LD·ATTECs Increased Thermogenesis in db/db Mice

**Fig. 3:**
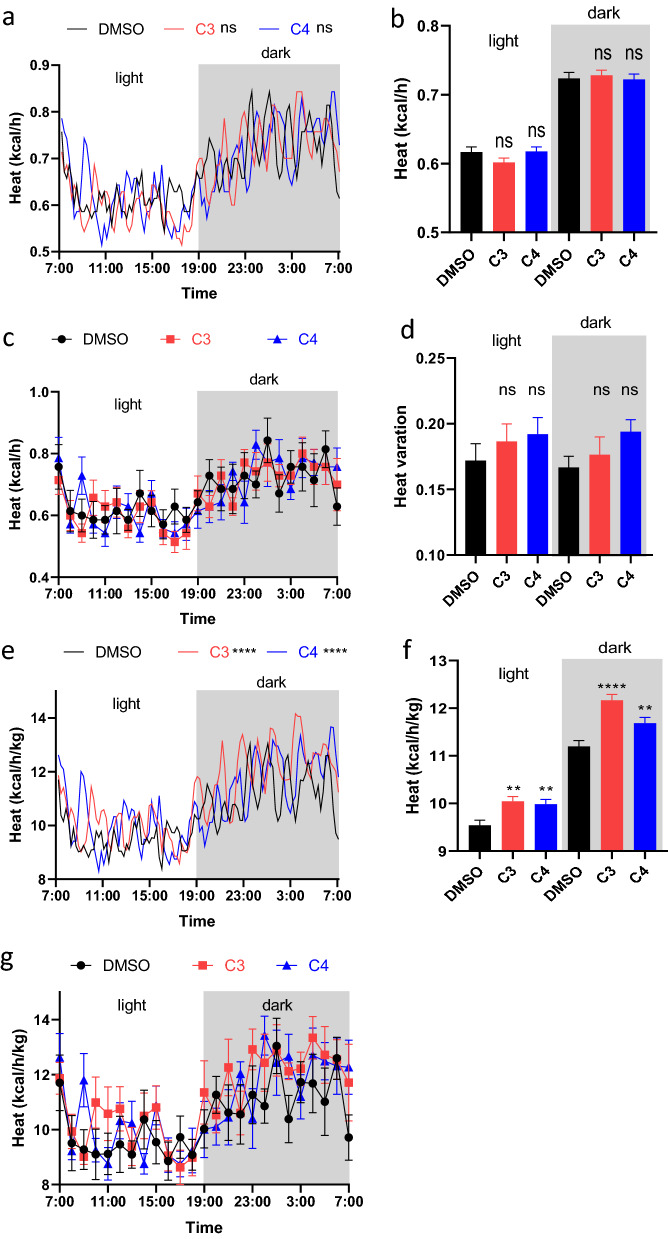
LD·ATTECs affect thermogenesis phenotypes in *db/db* mice. **a**, **b** Whole-body thermogenesis of mice during the day phase (12 h) and dark phase (12 h) (all data points). **c** Whole-body thermogenesis of mice during the day phase (12 h) and dark phase (12 h) (hourly data points). **d** Coefficient of variation analysis individual thermogenesis. **e**, **f** Whole-body thermogenesis (normalized to body weight) of mice during the day phase (12 h) and dark phase (12 h) (all data points). **g** Whole-body thermogenesis (normalized to body weight) of mice during the day phase (12 h) and dark phase (12 h) (hourly data points). *N* = 7 per group, data are mean ± SEM. **p value < 0.01; ****p value < 0.0001; ns, *p* value > 0.05. One-way ANOVA (**b**, **d**, **f**), two-way ANOVA (**a**, **e**)

Thermogenesis is a key parameter in energy balance. The metabolic cage experiment data demonstrated that C3 or C4-injected mice showed no significant change in the whole-body thermogenesis **(**Fig. 3a–c). Meanwhile, compared with the DMSO-injected control, the coefficient of heat production variation of each individual mouse was only marginally increased in the C3 or C4-injected mice (Fig. 3d), indicating that LD·ATTECs may lead to non-significant fluctuated differences of thermogenesis in individual mouse. Since the body weights are different among different groups and the body weight may influence whole-body thermogenesis, we further normalized the whole-body thermogenesis to body weight to obtain more accurate comparisons. The data revealed that C3 or C4-injection increased thermogenesis after body weight normalization (Fig. 3e–g). The coefficient of variation of individual thermogenesis after normalization to body weight is the same as one before normalization in Fig. 3d; therefore, the coefficient variation of heat production is not shown. The increased thermogenesis suggested that higher energy consumption, which is consistent with higher oxygen consumption and carbon dioxide production.

### LD·ATTECs Influenced Exercise Activities in db/db Mice

**Fig. 4:**
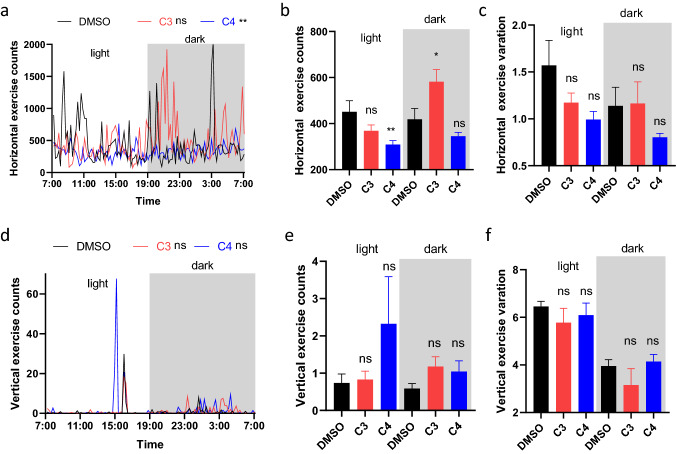
LD·ATTECs affect the movements in *db/db* mice. **a**, **b** Horizontal exercise counts during the day phase (12 h) and dark phase (12 h) (all data points). **c** Coefficient of variation analysis of individual horizontal exercise. **d**, **e** Vertical exercise counts during the day phase (12 h) and dark phase (12 h) (all data points). **f** Coefficient of variation analysis of individual vertical exercise. *N* = 7 per group, data are mean ±  SEM. *p value < 0.05; **p value < 0.01; ns, *p* value > 0.05. One-way ANOVA (**b**, **c**, **e**, **f**), two-way ANOVA (**a**, **d**)

In order to investigate whether LD·ATTECs influence the exercise, we monitored the mouse exercise in the horizontal and vertical directions during the light and dark phases with video and performed quantitative analyses. The C3-injection enhanced the horizontal exercise during the dark phase, but marginally decreased it during the light phase (Fig. 4a, b); we noted that these *db/db* mice’ exercise was almost identically between light and dark phases, and this might be related with the *db/db* mutation and the metabolic cage experimental condition. Under this condition, the C4-injected mice exhibited significantly decreased horizontal exercises compared with the DMSO control (Fig. 4a), especially during the light phase (Fig. 4b). Besides, compared with the control, the coefficients of horizontal exercise variation of individual mice showed the unchanged (C3-injected mice in dark phase) or decreased (C3-injected mice in light phase or C4-injected mice in both phases) trend for C3 or C4-injected mice (Fig. 4c), indicating that C3 or C4-injected mice had a unchanged degree of horizontal exercise fluctuation over the test period. Meanwhile, compared with the control group, the vertical exercise was slightly increased in mice injected with compound C3 or C4 (Fig. 4d, e; C3-injected mice in dark phase and C4-injected mice in both phases), although the changes were not statistically significant. The coefficient of vertical exercise variation was not significantly changed (Fig. 4f). Collectively, a slightly changed trend of horizontal and vertical exercises by the injection of LD·ATTECs was observed (Fig. 4b, e), possibly providing partial contribution to the elevated energy consumption (Fig. 3e–g).

### LD·ATTECs Reduced Blood Glucose Levels in db/db Mice

**Fig. 5:**
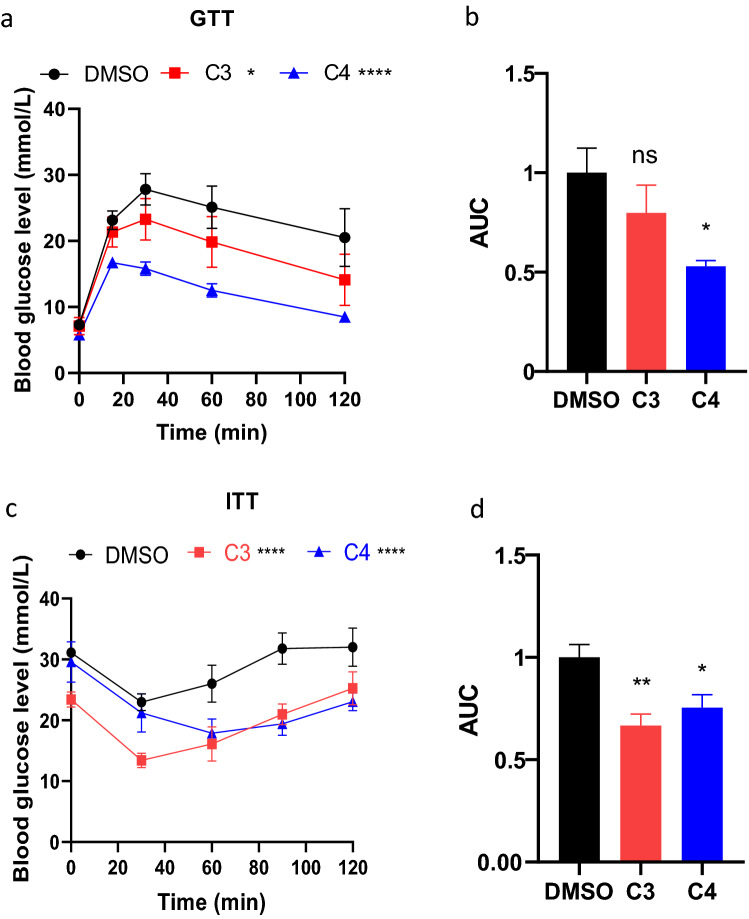
LD·ATTECs improved the glucose homeostasis in the *db/db* mouse model. **a** The blood glucose level during glucose tolerance test. **b** Area under the curve (AUC) of the blood glucose level of GTT. **c** The blood glucose level during insulin tolerance test. **d** Area under the curve (AUC) of the blood glucose level of ITT. *N* = 7 per group (except *N* = 6 for DMSO and C4 groups in ITT assay), data are mean ±  SEM. *p value < 0.05; **p value < 0.01; ****p value < 0.0001; ns, *p* value > 0.05. One-way ANOVA (**b**, **d**), two-way ANOVA (**a**, **c**)

Abnormal blood glucose is a major pathogenic symptom associated with obesity, and the *db/db* mice displayed markedly elevated glucose level in the glucose tolerance tests (GTT) (Zhang and Macielag 2020). To evaluate the effect of LD·ATTECs on blood glucose levels, we performed GTT in mice 14 days after the compound treatment. For GTT, mice received an intraperitoneal injection of glucose (2 g/kg body weight) after fasting for overnight, and the tail blood glucose levels were measured at different time points. The C3 or C4-injected mice exhibited lower blood glucose levels and stronger glucose tolerance than the ones exhibited in the DMSO-injected control group (Fig. 5a, b), indicating that the reduction of LDs improves blood glucose in the *db/db* mouse model. In addition to GTT, we did insulin tolerance test (ITT) using 10-month-old BKS-db mouse (BKS-Lepr^em2Cd479^/Gpt), and we found that the C3 or C4-injected mice had improved insulin sensitivity than that of DMSO-injected control group (Fig. 5c, d). Collectively, LD·ATTECs improved glucose homeostasis in the *db/db* mouse model.

**Fig. 6:**
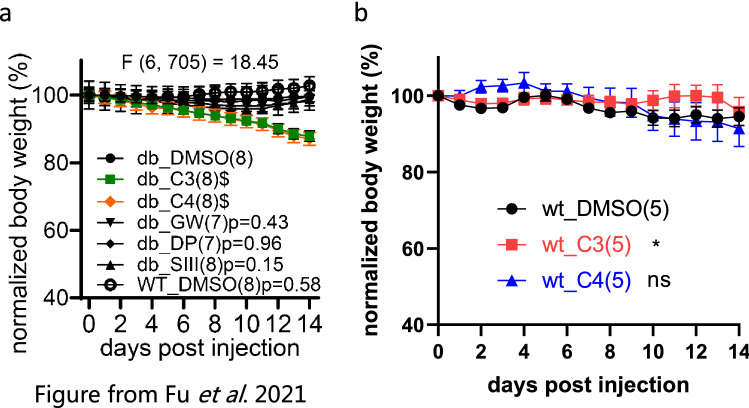
LD·ATTECs alter phenotypes of *db/db* mice via LD degradation. **a** Measurements of body weight (measured each day and normalized to the averaged weight of day 0) in the *db/db* mice injected with LD·ATTECs from Fu et al. (2021). The replicate number indicates the number of mice, ^$^*p* value < 0.0001, exact *p* values are shown in figure. **b** Measurements of body weight (measured each day and normalized to the averaged weight of day 0) in the wild-type mice injected with LD·ATTECs. *N* = 5 per group (one mouse died in the C3 group on day six, whose body weight was used as a constant value for the next eight days record), data are mean ±  SEM. *p value < 0.05; ns, *p* value > 0.05. Two-way ANOVA (**a**, **b**)

**Fig. 7:**
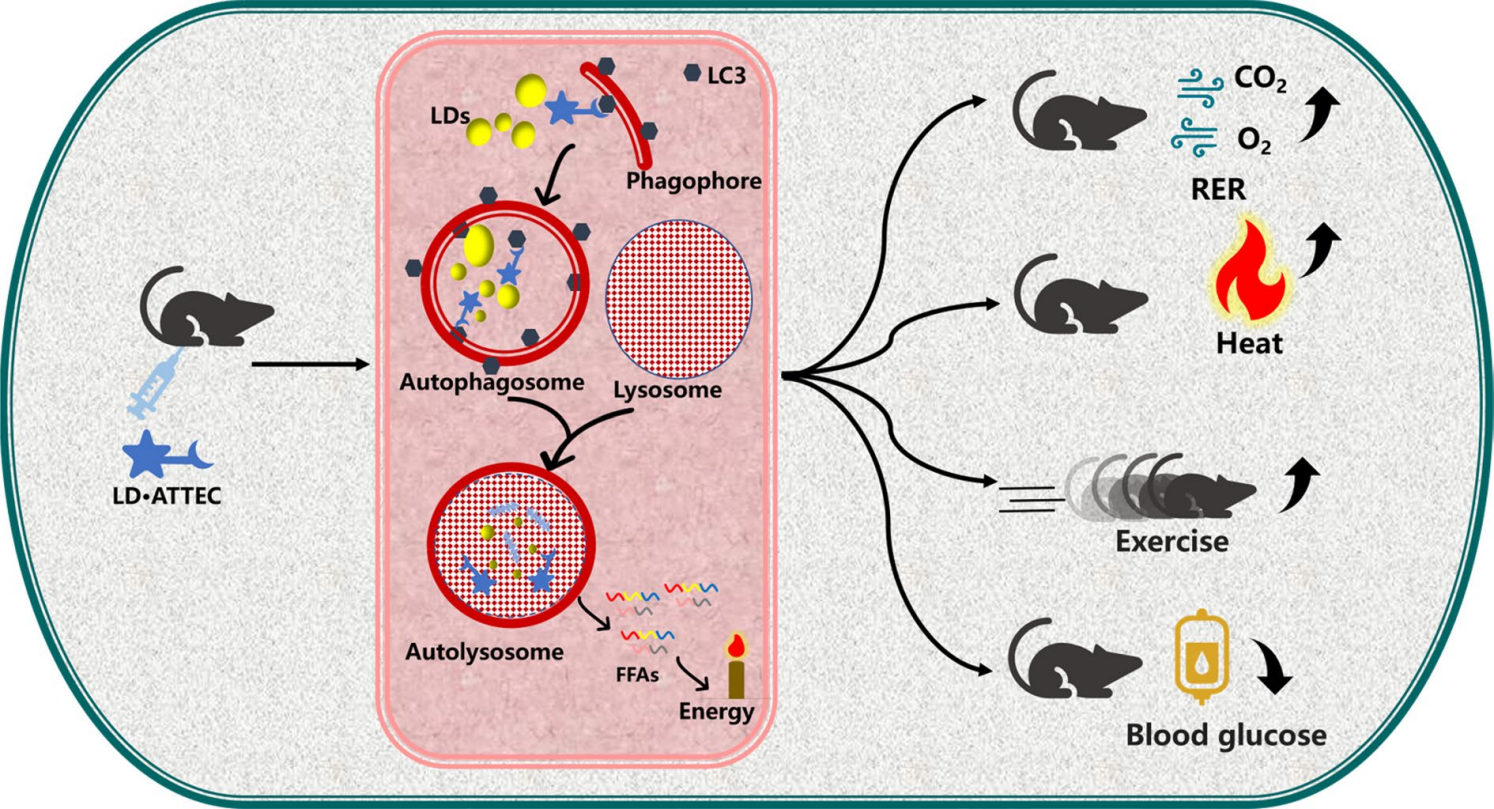
Autophagic clearance of LDs alters metabolic phenotypes in a *db/db* mouse model. LD·ATTECs could enhance lipophagy and improve the metabolic phenotypes of *db/db* mice. LD·ATTECs increased the uptake of oxygen and the release of carbon dioxide, enhanced the heat production, partially enhanced exercise, and decreased the blood glucose level. Combined with our previous published reports, LD·ATTECs pull LDs to autophagosomes and then to lysosomes for degradation and utilization

Taken together, our study characterized the metabolic phenotype changes induced by LD·ATTECs in the *db/db* mouse model. The clearance of LDs by LD·ATTECs could enhance  the respiration, thermogenesis, and influenced exercise, and it also decreased blood glucose. The overall trend is towards correcting the metabolic abnormalities in the *db/db* mice, suggesting that the decrease of LDs accumulation by autophagic degradation could be beneficial at the phenotypic level. To validate if LD·ATTECs is able to improve the metabolic phenotype of *db/db* mice by decreasing the LDs accumulation, we published that LD·ATTECs can reduce the body weight of *db/db* mice (Fig. 6a) (Fu et al. 2021); here, we injected the wild-type mouse with LD·ATTECs and confirmed that LD·ATTECs did not reduce the body weight of wild-type mice (Fig. 6b), and the results indicated that LD·ATTECs reduced *db/db* mouse body weight was not due to compound toxicity. Taken together, the model of LD·ATTECs improves the phenotype of *db/db* animals, which is described as Fig. 7.

## Discussion

Accumulation of LDs contributes to obesity–diabetes progression (Gluchowski et al. 2017; Kojta et al. 2020). Previous studies showed lipids accumulation in the kidneys of diabetic patients and experimental animals (Lee et al. 1991). To study whether harnessing LD·ATTECs can improve metabolic phenotype in obesity–diabetes, in this study, we synthesized and used LD·ATTECs to reduce cellular LDs accumulation in *db/db* mice. Compared with the control, C3 or C4-injected mice showed higher thermogenesis and healthier respiratory phenotype (Figs. 1, 2, 3). Together, our study demonstrated that LDs clearance contributes to improve metabolic phenotype in *db/db* mouse model. Our results are consistent with the previous studies that reduction of LDs accumulation can alleviate disease in the *db/db* mouse model (Jo et al. 2021; Lee et al. 2016; Liu et al. 2018). However, the extent to which the degradation of redundant LDs improves diabetes requires further study.

Obesity produces hyperglycemia and hyperlipidemia, both of which contribute to the progression of diabetes, and a process is involved with disturbed metabolism. Lipophagy is a process of selective degradation of LDs by autophagy, which provides energy for maintaining energy homeostasis, but lipophagy is dysregulated in *db/db* mice (Han et al. 2021; Korolenko et al. 2022); as a result, energy imbalance exacerbated obesity–diabetes. In our study, we found that autophagic clearance of LDs by LD·ATTECs enhanced O_2_ consumption and CO_2_ production (Fig. 1), and the body weight normalized energy consumption (Fig. 3), possibly contributed partially by enhanced exercises during the dark phase (Fig. 4). Meanwhile, the changes in exercises alone may not explain all the energy consumption changes, because the changes were marginal and the changes of exercise during the light phase were somewhat complicated (Fig. 4). Besides altered exercises, the enhanced basal metabolism may also contribute to increase heat production. LD·ATTECs can decrease fat-to-lean ratio (Fu et al. 2021), and the LD·ATTECs increased browning of white adipose tissue (WAT) may also contribute to the heat production (data not shown). Therefore, we cautiously believe that the degradation of LDs contributes to higher thermogenesis and lower blood glucose level, both benefiting from the degradation and utilization of LDs. Maintaining high whole-body energy expenditure can protect mice from diet-induced obesity and improve diabetes, and LD·ATTECs increased lipid degradation and energy expenditure in our study.

RER was calculated as the ratio of CO_2_ to O_2_ (Choi et al. 2015), which indicates carbohydrate or fat utilization. Many previous studies showed that *db/db* mice had lower RER compared to wild type (Choi et al. 2015; Osborn et al. 2010), indicating that *db/db* mice has metabolic defects. The C3 or C4-injected mice reversed defects and displayed higher RER compared with control mice, and LD·ATTECs increased thermogenesis (Fig. 3).

In addition to the above phenotypes, we observed that LD·ATTECs can reduce body weight and the lipids in both liver and serum without affecting calorie intake in our previous study (Fu et al. 2021), and the FFA resulted from LDs degradation is utilized via beta-oxidation . Therefore, we concluded that LDs degradation plays a key role in improving the phenotype via FFA utilized. Because of no ATG5 KO mice were used to metabolic cages assay, we cannot fully assess the effects of none autophagy on our observed phenotypes, but we validated the autophagy dependence of LDs degradation in ATG5 KO cell models and other autophagy-dependent assay in previous study (Fu et al. 2021). Besides, LD·ATTECs did not reduce the body weight of wild type (Fig. 6b), and wild mice has much lower LDs, so body weight changes were caused via LDs degradation rather than off-site toxicity in the *db/db* model. Therefore, we further believe that the improvement of phenotype in the model mice is the result of the degradation and utilization of LDs by the autophagy pathway. LD·ATTECs promoted LDs utilization and increased the lean-fat ratio, and the browning of adipose tissue may contribute to phenotypes; however, we cannot exclude exercise fluctuation that may promote thermogenesis and respiratory in turn. Nonetheless, we need further study on the relationship between exercise, LDs degradation, and metabolism.

Conclusively, our findings showed that LD·ATTECs improve metabolic phenotypes in vivo, and we revealed the novel functional impacts of autophagic clearance of LDs via chemical biology strategy, providing insights into LD biology and obesity–diabetes pathogenesis from the phenotypic perspective.

## Conclusions

Here, the study validated LD·ATTECs’ effects on metabolic phenotypes in mouse models. The study characterized the metabolic phenotypes improved by LD·ATTECs in an obesity–diabetes mouse model, such as increased the oxygen uptake and the release of carbon dioxide, enhanced the heat production of animals and slightly enhanced the exercise during the dark phase, decreased the blood glucose level and improved insulin sensitivity. Collectively, the study revealed novel functional impacts of autophagic clearance of LDs in obesity–diabetes pathogenesis from the phenotypic perspective.

## Data Availability

The datasets generated and material used for this study are available upon request from the corresponding authors.
